# Structural Variant and Repeat Expansion Findings Identified by Optical Genome Mapping in Complex Autism Spectrum Disorder With Concomitant Neurodevelopmental Disorders

**DOI:** 10.1155/humu/3130383

**Published:** 2026-06-11

**Authors:** Mehmet Burak Mutlu, Özge Beyza Gündoğdu Öğütlü, Özlem Öz, Fahrettin Duymuş, Serhat Seyhan, Ayşe Gül Bayrak Tokaç, Esad Tezcan, Hakan Öğütlü, Fatma Demiryılmaz, Nurcan Silahtarlıoğlu, Kader Bilgil, Sümeyye Elma, Murat Erdoğan, Hakan Gümüş, Sefer Kumandaş, Fethiye Kılıçaslan

**Affiliations:** ^1^ Detagen Genetic Diseases Evaluation Center, Kayseri, Türkiye; ^2^ Department of Medical Genetics, Erzurum Regional Training and Research Hospital, Erzurum, Türkiye, erzurumbeah.gov.tr; ^3^ Department of Medical Genetics, Harran University Faculty of Medicine, Şanlıurfa, Türkiye, harran.edu.tr; ^4^ Department of Medical Genetics, Uşak University, Faculty of Medicine, Uşak, Türkiye, usak.edu.tr; ^5^ Medroyal Genetic Diseases Assessment Center, İstanbul, Türkiye; ^6^ Department of Internal Medicine, Division of Medical Genetics, İstanbul University, Faculty of Medicine, İstanbul, Türkiye, istanbul.edu.tr; ^7^ Department of Child and Adolescent Psychiatry, Selçuk University, Faculty of Medicine, Konya, Türkiye, selcuk.edu.tr; ^8^ Department of Child and Adolescent Psychiatry, University College Dublin, Dublin, Ireland, ucd.ie; ^9^ Department of Medical Genetics, University of Health Science, Kayseri Faculty of Medicine, Kayseri, Türkiye, sbu.edu.tr; ^10^ Department of Pediatrics, Division of Pediatric Neurology, Erciyes University, Faculty of Medicine, Kayseri, Türkiye, erciyes.edu.tr; ^11^ Department of Child and Adolescent Psychiatry, Harran University, Faculty of Medicine, Şanlıurfa, Türkiye, harran.edu.tr

**Keywords:** autism spectrum disorder, copy-number variants, cytogenomics, neurodevelopmental disorders, optical genome mapping, repeat expansion, structural variants

## Abstract

Autism Spectrum Disorder (ASD) is a neurodevelopmental disorder characterized by persistent deficits in social communication and interaction, along with restricted, repetitive patterns of behavior, interests, or activities. Single‐nucleotide variants (SNVs) and structural variants (SVs), including copy‐number variants (CNVs), have been reported as important contributors to the genetic basis of ASD. In this study, we evaluated the diagnostic contribution of optical genome mapping (OGM) as a complementary cytogenomic approach in a selected ASD cohort enriched for complex ASD with developmental delay/intellectual disability (DD/ID) and/or additional neurodevelopmental or syndromic features. We retrospectively evaluated 34 individuals with ASD who underwent OGM analysis, most of whom had concomitant DD/ID and/or additional neurological, congenital, or syndromic features. Confirmed pathogenic (P) or likely pathogenic (LP) findings were identified in 7/34 individuals (20.6%), and one additional unconfirmed OGM‐only duplication was provisionally interpreted as pathogenic. Overall, confirmed or provisional P/LP findings were observed in 8/34 individuals (23.5%), all of whom had complex ASD with DD/ID and/or additional neurodevelopmental or syndromic features. OGM‐detected findings were confirmed by orthogonal methods whenever clinically and technically feasible, and segregation analyses were performed when samples were available. These results suggest that OGM may add value to integrated genetic testing workflows for selected individuals with complex ASD, particularly when SVs, complex chromosomal rearrangements, or *FMR1* full‐mutation repeat expansions are clinically suspected or insufficiently resolved by conventional approaches.

## 1. Introduction

Autism Spectrum Disorder (ASD) is a neurodevelopmental disorder (NDD) characterized by persistent deficits in social communication and interaction, along with restricted, repetitive patterns of behavior, interests, or activities [1]. The pooled prevalence of ASD has been estimated at 0.72%, although estimates vary across studies, regions, and time periods [2, 3]. ASD is a leading cause of disability in children under five and imposes substantial economic, emotional, and physical burdens on families and society [4, 5].

Genetic factors contribute significantly to the etiology of ASD, with heritability estimates ranging from 40% to 90% based on evidence from sibling and twin studies [6]. Genetic studies have shown that approximately 20%–35% of ASD cases may be attributable to rare genetic variants, including single‐nucleotide variants (SNVs) and structural variants (SVs), particularly copy‐number variants (CNVs) [7, 8]. Non‐CNV SVs, including balanced chromosomal rearrangements such as inversions and cryptic translocations, have also been implicated in ASD, although they are less frequently detected by conventional methods [9, 10].

The diagnostic yield of whole exome sequencing (WES) and chromosomal microarray analysis (CMA) for identifying genetic contributors to ASD is highly variable and depends on cohort selection, phenotype complexity, and analytic strategy. In a cohort of 405 individuals with ASD, WES yielded diagnostic findings in 13.1% of individuals for SNVs/indels, with an additional 3.2% yield from exome‐based CNV analysis, resulting in a combined yield of approximately 16% [11]. Another clinical cohort reported a higher diagnostic yield for WES than for CMA, 24.5% versus 6.2%, respectively [12].

The 2020 American Academy of Pediatrics (AAP) guidelines continue to recommend CMA and Fragile X testing as part of the initial diagnostic workup for ASD, with exome sequencing considered only after negative results from these tests, and *MECP2* testing if relevant [13]. The role of CMA as a first‐tier test has been increasingly debated in some centers because of its lower diagnostic yield compared with newer approaches such as WES and genome sequencing [14]. However, none of these methods is sufficient to capture the full spectrum of genetic variation associated with ASD; therefore, many cases remain unexplained. These limitations highlight the need for complementary genomic technologies that may improve diagnostic resolution in selected cases.

The available genomic approaches differ in the classes of variants they detect. CMA is useful for genome‐wide CNV detection but cannot identify balanced rearrangements or repeat expansions, whereas WES mainly detects coding SNVs and small indels and has limited sensitivity for complex SVs and repeat expansions. Short‐read WGS and long‐read sequencing broaden variant detection, but their clinical implementation and SV interpretation remain dependent on platform availability, bioinformatic pipelines, and validation strategies. OGM therefore represents a complementary cytogenomic approach for detecting genome‐wide SVs, CNVs, balanced rearrangements, aneuploidies, and selected repeat expansions, while remaining limited for SNVs and small indels.

Optical genome mapping (OGM) is an emerging, probeless, culture‐free, and PCR‐free cytogenomic technology that may complement existing genetic testing approaches by enabling genome‐wide detection of SVs and CNVs in individuals with ASD and other NDDs [15]. Compared with CMA, OGM provides additional cytogenomic information by detecting several classes of SVs, including balanced rearrangements such as inversions and translocations, while also enabling genome‐wide CNV assessment [16]. OGM has shown 98% concordance with CMA for CNV detection and can refine genomic architecture while identifying clinically relevant SVs that may be missed by CMA [17]. OGM provides uniform genome‐wide coverage and may streamline laboratory workflows, potentially reducing turnaround time [18]. OGM may help address selected diagnostic gaps left by conventional cytogenetic and sequence‐based approaches, particularly for complex chromosomal rearrangements and SVs that are difficult to resolve using standard methods.

Therefore, the present study is aimed at evaluating the contribution of OGM in a clinically characterized cohort of individuals with ASD, most of whom had accompanying developmental delay/intellectual disability (DD/ID) and/or additional neurological, congenital, or syndromic features. The OGM workflow used in this study represents an established analytical approach rather than a new methodology. The value of this study lies in the ASD‐focused clinical framework, the characterization of a Turkish cohort, and the identification of rare CNVs, chromosomal rearrangements, aneuploidy, and *FMR1* full mutation repeat‐expansion findings within a single cytogenomic workflow. We therefore position OGM as a complementary method to exome/genome sequencing rather than as a replacement for sequence‐based testing.

## 2. Methods

### 2.1. Participants and Procedures

During the study period (2022–2025), 34 individuals with a clinical diagnosis of ASD who underwent OGM analysis were assessed for eligibility. Individuals were eligible for inclusion if they met all of the following criteria: (i) a clinical diagnosis of ASD established by child and adolescent psychiatrists according to DSM‐5 criteria; (ii) evaluation in the participating child psychiatry and/or medical genetics clinics during the study period; (iii) availability of OGM data generated from peripheral blood‐derived genomic DNA; and (iv) availability of sufficient clinical information for phenotype review. Individuals were excluded if the ASD diagnosis could not be confirmed from medical records, if OGM analysis failed to meet predefined quality‐control metrics, or if clinical data were insufficient for interpretation. All 34 eligible individuals met these criteria and were included in the final analysis; no eligible individual was excluded after medical record review. Because this was a retrospective study based on existing clinical and laboratory records, there was no active recruitment process and no refusal rate was applicable. Medical records from the participating child psychiatry and medical genetics clinics were retrospectively reviewed. ASD diagnoses were established by child and adolescent psychiatrists according to DSM‐5 criteria. Demographic data, developmental history, intellectual functioning, psychiatric comorbidities, neurological/systemic findings, and dysmorphology assessments were extracted from hospital records. Dysmorphology assessments were performed by clinical geneticists. The study protocol was approved by the Harran University Clinical Research Ethics Committee (HRÜ/24.14.22). Written informed consent for genetic testing and the use of anonymized clinical and genomic data for research and publication was obtained from the parents or legal guardians of all participants. All data were anonymized before analysis and manuscript preparation. OGM‐detected findings were confirmed using orthogonal methods whenever clinically and technically feasible. Findings that could not be confirmed by an alternative method were explicitly indicated as unconfirmed and interpreted cautiously.

For phenotypic stratification, individuals were categorized as having isolated ASD or complex ASD. Isolated ASD was defined as ASD without documented DD/ID, major congenital anomalies, epilepsy, dysmorphic features, or other neurological/systemic findings. Complex ASD was defined as ASD accompanied by DD/ID and/or additional neurological, congenital, dysmorphic, or systemic features. DD was defined as a significant delay in one or more developmental domains in children for whom a formal cognitive assessment was not available or age‐appropriate. ID was defined based on documented clinical assessment of impaired intellectual and adaptive functioning; when available, IQ scores were recorded.

### 2.2. OGM

Peripheral blood samples were collected in EDTA tubes, and ultrahigh molecular weight DNA was isolated according to the manufacturer′s protocol using the Bionano Prep SP‐G2 Fresh Human Blood DNA Isolation protocol. DNA quality and concentration were assessed before labeling. Direct Label and Stain chemistry was performed using the Bionano Prep DLS‐G2 protocol. Labeled DNA molecules were loaded onto Saphyr chips and imaged on the Saphyr instrument (Bionano, San Diego, California, United States).

Raw molecule data were processed using Bionano Solve V3.8.2. De novo assembly was performed for genome‐wide SV detection, including CNV analysis, and the EnFocus Fragile X pipeline was used for *FMR1* repeat‐expansion analysis. SV and CNV calls were visualized and manually reviewed using Bionano Access V1.8.2. Analyses were performed using the GRCh38/hg38 reference genome. Sample‐level quality‐control thresholds included map rate ≥ 70%, label density 14–17 labels/100 kbp, filtered molecule N50 ≥ 150 kbp for molecules ≥ 20 kbp, and N50 ≥ 230 kbp for molecules ≥ 150 kbp.

Reportable variants were prioritized based on size, gene content, overlap with known disease‐associated regions or dosage‐sensitive genes, inheritance pattern when available, phenotypic concordance, and absence or rarity in population/control datasets. CNVs were classified according to the ACMG/ClinGen technical standards for constitutional CNV interpretation [19]. For balanced rearrangements, numerical chromosome abnormalities and repeat expansions, the ACMG/ClinGen CNV scoring framework was not directly applicable; therefore, classification was based on established disease association, event type, genomic content or breakpoint involvement, phenotype concordance, inheritance/segregation data when available, and orthogonal confirmation. Clinically relevant OGM findings were confirmed by CMA, karyotype, WES‐CNV, WGS, or fragment analysis whenever technically and clinically feasible.

## 3. Results

Of the 34 individuals included in the study, 30 had documented DD/ID, frequently accompanied by additional neurodevelopmental, neurological, congenital, dysmorphic, or systemic findings, whereas 4 had ASD without documented DD/ID, although some had additional neurological findings such as epilepsy, infantile spasms, spastic paresis, or gait abnormality. All confirmed or provisional pathogenic/likely pathogenic (P/LP) findings were identified in individuals with complex ASD. No confirmed or provisional P/LP finding was detected among individuals with ASD without DD/ID or other major neurodevelopmental findings. Table 1 summarizes the sociodemographic and clinical features of participants with OGM‐detected findings. The corresponding data for the remaining participants are provided in Table S1.

**Table 1:** The sociodemographic information and clinical features of participants with OGM‐detected findings.

Participant	Sex	Age	Psychiatric features	Other clinical features
1	F	5	ASD, ADHD	DD/ID, speech delay, delayed ability to walk, learning disability, atrial septal defect, dysmorphic features (epicanthus inversus, frontal bossing, upslanting palpebral fissures)
2	M	9	ASD, aggressive behavior	DD/ID, speech delay, epilepsy, specific food allergy, hypermetropia
3	F	16	ASD	DD/ID, speech delay, inability to walk, ataxia, bilateral hearing loss
4	M	4	ASD	DD/ID, hypotonia, inflammatory intestinal disease
5	M	5	ASD (with Asperger‐like features), OCD	Specific food allergies, dysphagia
6	F	3	ASD	DD/ID, speech delay
7	F	3	ASD	DD/ID, speech delay, delayed ability to walk, bilateral hearing loss, leukocytosis, delayed myelination on MRI
8	M	5	ASD	DD/ID, hypotonia, gait difficulty, unilateral testicular atrophy (right) and agenesis (left), micropenis, hypogonadotropic hypogonadism
9	M	11	ASD	DD/ID, speech delay, delayed ability to walk, myopia, brachydactyly, short stature, scoliosis, obesity, local alopecia
10	F	3	ASD, happy personality, stereotypic movements	DD/ID, inability to speak and walk, epilepsy, microcephaly, dysmorphic features, ptosis, strabismus
11	F	4	ASD, stereotypic movements	DD/ID, inability to speak and walk, hypotonia, strabismus, epilepsy
12	M	9	ASD	DD/ID, speech delay, delayed ability to walk, hypotonia, epilepsy, microcephaly
13	M	2	ASD, happy personality	DD/ID, speech delay, delayed ability to walk, epilepsy, oculocutaneous albinism, arthrogryposis, craniosynostosis, macroorchidism, long face, large ears

Abbreviations: ADHD, attention deficit hyperactivity disorder; ASD, autism spectrum disorder; DD/ID, developmental delay/intellectual disability; F, female; M, male; OCD, obsessive compulsive disorder.

Overall, OGM identified clinically relevant rare genomic findings in 13 of 34 participants (38.2%), whereas 21 participants had no reportable rare OGM finding. At the participant level, 11 individuals had a single clinically relevant OGM finding, whereas 2 individuals had multiple related findings: P1 had an unbalanced translocation with associated 5q35.3 deletion and 22q13.31q13.33 duplication, and P13 had *FMR1* repeat expansion with two insertion calls suggestive of mosaicism. Across the cohort, the reported findings included CNVs in 10 participants, translocations in 2 participants (1 balanced and 1 unbalanced), a numerical sex chromosome abnormality in 1 participant, and an *FMR1* full‐mutation repeat expansion in 1 participant. Most findings were nonrecurrent at the locus/event level; however, two participants had rare deletions involving the 7p22.3 region. Seven participants had confirmed P/LP findings, and one additional participant had an unconfirmed OGM‐only duplication interpreted as provisional pathogenic. Five participants had variants of uncertain significance (VUS). Of the 13 participants with OGM‐detected findings, 11 had findings confirmed by orthogonal methods. Two findings, the 9q34.11q34.2 duplication in P4 and the 21q11.1q21.1 duplication in P12, could not be confirmed by an alternative technology and are therefore indicated as unconfirmed in Table 2. The OGM analysis results, orthogonal confirmation status, segregation analysis, and diagnostic interpretation are summarized in Table 2.

**Table 2:** The OGM analysis results, confirmation methods, segregation analysis, and diagnostic interpretation of participants.

Participant No.	1	2	3	4	5	6	7	8	9	10	11	12	13
OGM	OGM [GRCh38] t(5;22)(q35.3;q13.31); OGM [GRCh38] 5q35.3 (177762086_181472714) × 1; OGM [GRCh38] 22q13.31q13.33 (45271390_50805587) x3	OGM [GRCh38] 16p13.2 (8890564_8932865) x1	OGM [GRCh38] 6q14.3 (85502573_85575125) x0	OGM [GRCh38] 9q34.11‐q34.2 (129590783_133526602) x3	OGM [GRCh38] 7p22.3 (2510943_2553878) x1	OGM [GRCh38] t(8;11)(q11.21;p13)	OGM [GRCh38] 7p22.3 (2223834_2299992) x1	OGM [GRCh38] Xp22.33‐q28 (1480887_156025612) x4	OGM [GRCh38] 15q24.3 (76463787_76473525) x0	OGM [GRCh38] 18q21.2 (55239354_55333049) x1	OGM [GRCh38] 2q22.1q22.2 (141303678_141674411) x1	OGM [GRCh38] 21q11.1q21.1 (12406577_18258363) x3	OGM [GRCh38] ins(X;?)(q27.3;?)(147910127_147919379;?) OGM [GRCh38] ins(X;?)(q27.3;?)(147910189_147919317;?) OGM [GRCh38] ins(X;?)(q27.3;?) (147910189_147919317
Confirmation	CMA, karyotype	WES‐CNV	WES‐CNV	Not confirmed	WES‐CNV	Karyotype	WES‐CNV	Fragment analysis	WES‐CNV	WGS	CMA	Not confirmed	Fragment analysis
Size	Del: 3,710,629 bp	Del: 36,297	Del: 65,653 bp	Dup: 3,935,820 bp	Del: 27,527 bp	—	Del: 57,515 bp	Pentasomy	Del: 5527 bp	Del: 87,573 bp	Del: 345,944 bp	Dup: 5,851,786 bp	Repeat number: ~ 254 RU
	Dup: 5,534,198 bp												
Zygosity	Heterozygous	Heterozygo us	Homozygous	Heterozygous	Heterozygous	Heterozygous	Heterozygous	—	Homozygous	Heterozygous	Heterozygous	Heterozygous	Hemizygous, mosaic
Pathogenicity	P (Del 5q35.3: 1A, 2G, 2H, 3C, 4 L)	P (1A‐2B‐2D‐3A‐4L‐5H)	LP (1A‐2B‐2D‐3A)	Provisional P (unconfirmed) (1A‐2H‐3C‐4L‐5H)	VUS (1A‐3A)	VUS	VUS (1A, 2A, 2B, 2C, 3A, 4A,	P	LP (1A‐2B‐2E‐3A)	P (1A‐2B‐2E‐3A‐4L)	VUS (1A‐2H‐3A‐4O)	VUS (1A‐2H‐3A‐4L)	P
	P (Dup 22q13.31‐13.33: 1A, 2H, 3C, 4 L, 4 N)												
Inheritance	Maternal	Unknown	Paternal and maternal	Unknown	Paternal	Unknown	Unknown	—	Unknown	Unknown	Unknown	Unknown	Maternal
Diagnosis	5q35.3 Subtelomeric deletion syndrome	Hao‐Fountain syndrome	Autosomal recessive spinocerebellar ataxia 20	Possible 9q34 duplication syndrome	—	—	—	49,XXXXY syndrome	Intellectual developmental disorder and retinitis pigmentosa	Pitt‐Hopkins Syndrome	—	—	Fragile X syndrome
	22q13 Duplication Syndrome												

*Note:* The P4 duplication was not orthogonally confirmed; therefore, its classification and diagnostic interpretation should be considered provisional.

Abbreviation: CMA: chromosomal microarray, Del: deletion, Dup: duplication; LP: likely pathogenic, P: pathogenic, WES‐CNV: whole exome sequencing copy‐number variant analysis, WGS: whole genome sequencing, VUS: variant of uncertain significance.

## 4. Confirmed or Provisional P/LP Findings

**Figure 1:**
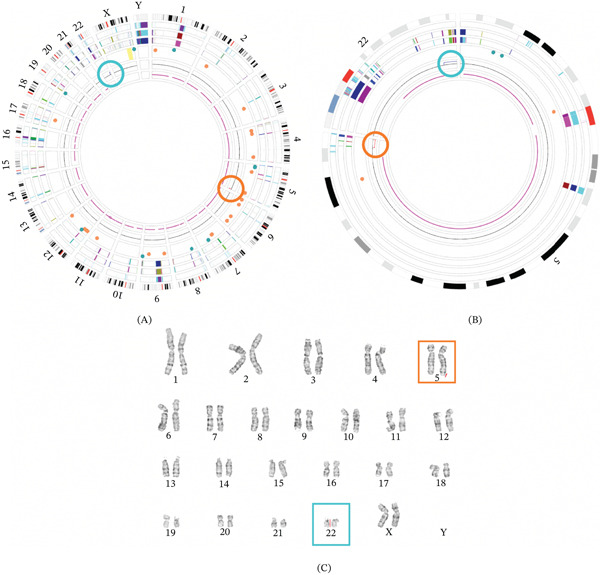
(A) The circos plot image of P1. The turquoise ring indicates the copy number gain in 22q13.31 and the orange ring indicates the copy number loss in 5q35.3. (B) Chromosome 5 and 22 are shown in the circos plot. The CNVs in 5q35.3 and 22q13.31 are shown in an enlarged view. (C) The karyotype of the participant′s mother. The turquoise rectangle indicates Chromosome 22 and the orange rectangle indicates Chromosome 5. The maternal karyotype shows a balanced t(5; 22)(q35.3; q13.31). The red line shows an additional fragment on the terminal region of Chromosome 5q and a slight downsize on Chromosome 22q.

In P1, OGM detected a 3,710,629‐bp deletion in 5q35.3 and a 5,534,198‐bp duplication in 22q13.31q13.33, consistent with an unbalanced translocation involving Chromosomes 5 and 22. Karyotype analyses were performed for both parents, and the rearrangement was found to originate from a maternal balanced translocation, t(5;22)(q35.3;q13.31). The 5q35.3 subtelomeric deletion is rare and has been associated with a phenotype characterized by DD, hypotonia, and distinctive craniofacial features, with *HNRNPH1* proposed as a candidate gene for psychiatric findings, including ASD, ADHD, and syndromic ID [20, 21]. In addition, a duplication in 22q13.31q13.33 associated with chromosome 22q13 duplication syndrome (OMIM#615538) was identified. This syndrome is characterized by DD, behavioral abnormalities including ASD, and other neuropsychiatric disorders, with *SHANK3* proposed as a major contributor [22]. The OGM results and the mother′s karyotype of P1 are shown in Figure 1.

**Figure 2:**
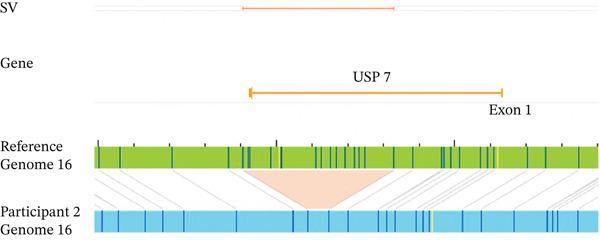
The genome browser image of P2. The deletion overlaps within *USP7* except Exon 1. Horizontal green and blue bars represent the reference and the participant genome of Chromosome 16, respectively. One red line in the SV track indicates the heterozygous variant.

In P2, OGM identified a 36,297‐bp heterozygous deletion in 16p13.2 involving *USP7*, resulting in the diagnosis of Hao‐Fountain syndrome (HAFOUS, OMIM#616863). This multiexonic deletion is predicted to disrupt the *USP7* coding sequence and result in a loss‐of‐function allele, most likely through nonsense‐mediated decay and/or production of a severely truncated nonfunctional protein. Individuals affected by HAFOUS mostly present with DD/ID, hypotonia, and characteristic behavioral phenotypes such as ASD, ADHD, aggressiveness, and compulsivity [23]. ASD is a common psychiatric manifestation of HAFOUS, supporting the phenotypic relevance of this deletion [24]. The OGM result of P2 is shown in Figure 2.

**Figure 3:**
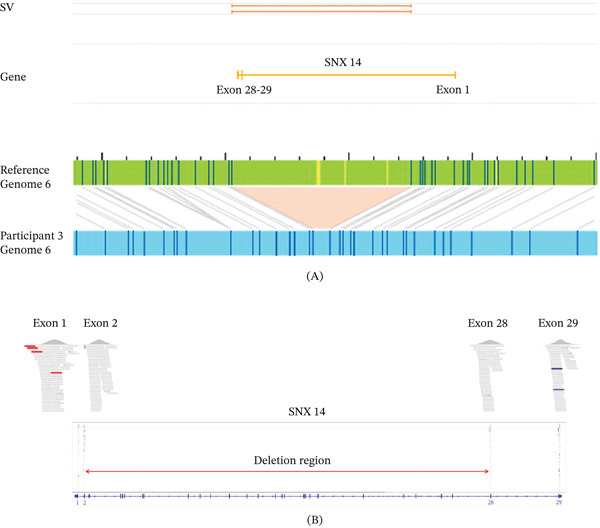
(A) The genome browser image of P3. The deletion overlaps within *SNX14*. The candidate region is 72,552 bp (covers the whole *SNX14* gene except Exon 1) and the deletion size is 65,653 bp in OGM. Horizontal green and blue bars represent the reference and the participant genome of Chromosome 6, respectively. Two red lines in the SV track indicate the homozygous variant. (B) The IGV image of P3. WES obtained the reads from Exons 1, 2, 28, and 29 of *SNX14*. The deletion is predicted to cover Exon 3, Exon 27, and in between. The red line presents the candidate deletion region (from Exon 2 to Exon 28). The blue line presents the exons of *SNX14*.

In P3, OGM identified a 65,653‐bp homozygous deletion in 6q14.3 involving *SNX14*, leading to the diagnosis of autosomal recessive spinocerebellar ataxia 20 (SCAR20; OMIM#616354). This homozygous multiexonic deletion is predicted to disrupt the *SNX14* reading frame and result in biallelic loss of function, most likely through nonsense‐mediated decay and/or production of a truncated nonfunctional *SNX14* protein. SCAR20 is a NDD with many features, including severe motor and cognitive DD, speech and gait abnormalities, coarse facial features, cerebellar atrophy, and ASD [25]. This SV was not detected by CMA, likely because of its size, platform resolution, and reporting filters. OGM identified the deletion, whereas WES‐CNV provided complementary exon‐level read‐depth information, indicating retained reads for Exons 1, 2, 28, and 29. These findings illustrate the complementary value of OGM and sequencing‐based CNV analysis for intragenic deletions. ASD is a prominent feature of SCAR20, which, to the best of our knowledge, is the only spinocerebellar ataxia subtype associated with ASD. Behavioral abnormalities, such as aggressiveness and lack of speech, have also been reported in individuals with *SNX14* mutations. SCAR20 should be placed in the differential diagnosis of cases of ASD with ataxia. The OGM and WES results of P3 are shown in Figure 3.

**Figure 4:**
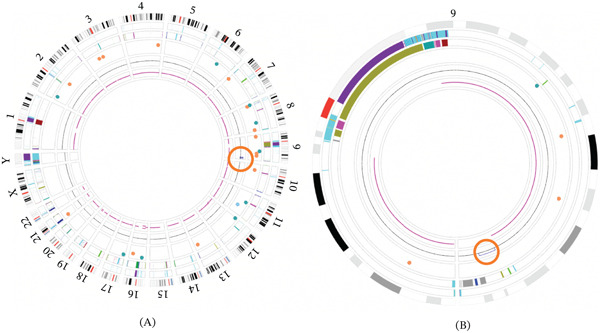
(A) The circos plot image of P4. The orange ring indicates the copy number gain in 9q34.11q34.2. (B) Chromosome 9 is shown in the circos plot. The copy number gain in 9q34.11q34.2 is shown in an enlarged view.

In P4, OGM detected a 3,935,820‐bp duplication in 9q34.11q34.2, which was interpreted as an OGM‐only finding consistent with chromosome 9q34 duplication syndrome. This duplication could not be confirmed by an alternative technology; therefore, its clinical interpretation was based on genomic size and content, phenotypic concordance, and overlapping pathogenic records in public databases, and should be considered provisional pending further validation. Although the duplicated interval in P4 was proximal to the commonly implicated 9q34.3 region, overlapping pathogenic records with similar clinical findings have been reported in public databases, including ClinVar ID: 59908 and DECIPHER patient 322988. In this region, *TSC1, TOR1A*, and *NTNG2* are candidate genes; however, there is no clear evidence linking duplications involving these genes to ASD [26–28]. The OGM results of P4 are shown in Figure 4.

**Figure 5:**
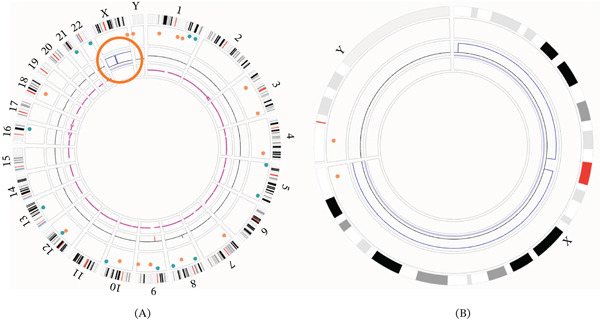
(A) The circos plot image of P8. The orange ring indicates the XXXX chromosome constitution. (B) Chromosome X and Chromosome Y are shown in the circos plot. The circos plot indicates the XXXXY chromosome constitution.

In P8, OGM detected a numerical sex chromosome abnormality consistent with 49,XXXXY syndrome. Although ASD has not been consistently reported in 49,XXXXY syndrome, language impairment, social communication difficulties, and ASD‐related features are frequently described in sex chromosome aneuploidies [29–31]. The OGM results of P8 are shown in Figure 5.

**Figure 6:**
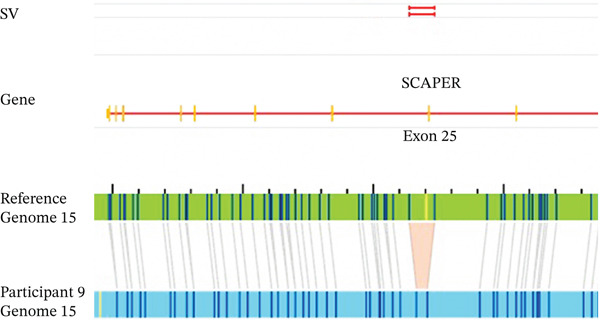
The genome browser image of P9. The deletion overlaps within *SCAPER*. The candidate region covers Exon 25. Horizontal green and blue bars represent the reference and the participant genome of Chromosome 15, respectively. Two red lines in the SV track indicate the homozygous variant.

In P9, OGM detected a novel 5,527‐bp homozygous deletion involving Exon 25 of *SCAPER* in 15q24.3, and the participant was diagnosed with intellectual developmental disorder and retinitis pigmentosa (IDDRP, OMIM#618195). This exon‐disrupting deletion is predicted to alter the *SCAPER* coding sequence and cause loss of function, either through nonsense‐mediated decay if a premature termination codon is generated or through production of a truncated nonfunctional protein. In IDDRP, behavioral problems have been described in about three‐quarters of published cases to date. Although ADHD is the most frequently reported behavioral finding, self‐injury, ASD, dyspraxia, and skin‐picking behavior have also been reported [32]. The OGM result of P9 is shown in Figure 6.

**Figure 7:**
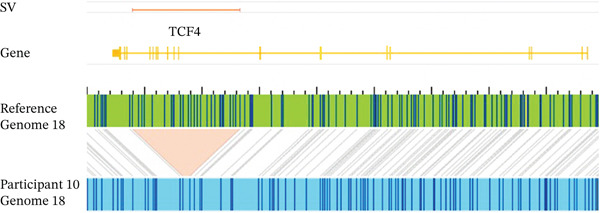
The genome browser image of P10. The deletion overlaps within *TCF4*. Horizontal green and blue bars represent the reference and the participant genome of Chromosome 18, respectively. The red line in the SV track indicates the heterozygous variant.

In P10, OGM detected an 87,573‐bp heterozygous deletion spanning Exons 9 through 15 of *TCF4*, and the participant was diagnosed with Pitt–Hopkins syndrome (OMIM#610954). This multiexonic deletion disrupts the *TCF4* coding sequence and is predicted to result in *TCF4* haploinsufficiency through nonsense‐mediated decay and/or production of a truncated nonfunctional protein. In addition, *TCF4* variants have been associated with neuropsychiatric disorders such as ASD, bipolar disorder, major depression, schizophrenia, and posttraumatic stress disorder [33]. The OGM result of P10 is shown in Figure 7.

## 5. Repeat Expansion

**Figure 8:**
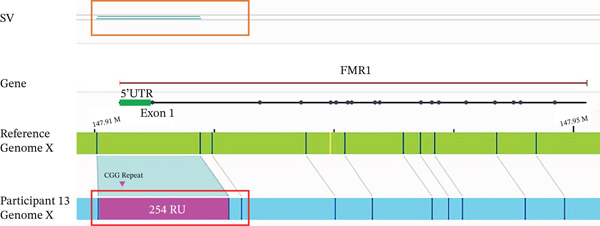
The genome browser image of P13. The insertions overlap with 5 ^′^ UTR (green bar), Exon 1, and Intron 1. The 5 ^′^ UTR of *FMR1* is the location of the characteristic repeat expansion of Fragile X syndrome. The purple box shows the 254 RU (repeat unit) in the repeat expansion region of *FMR1*. The horizontal green and blue bars represent the reference and participant genomes of Chromosome X, respectively. The black and red lines in the gene track represent the *FMR1* gene and its exon–intron structure. Two turquoise unequal lines in the SV track indicate the two different insertions.

In P13, the de novo assembly pipeline identified two insertion calls of approximately 9.1–9.3 kb in the *FMR1* repeat‐expansion region, whereas the EnFocus Fragile X pipeline estimated approximately 254 CGG repeats. The presence of two insertion calls with different breakpoints raised suspicion of mosaicism. Fragment analysis using two commercial kits confirmed full‐mutation‐range alleles with closely spaced repeat numbers, supporting *FMR1* mosaicism (Figure S1). Although the EnFocus pipeline estimated the overall full‐mutation range, it did not resolve the closely spaced mosaic alleles individually; combined interpretation with de novo assembly improved allele discrimination. The participant was diagnosed with Fragile X syndrome (OMIM#300624). In addition, OGM detected an insertion between Exons 18 and 19 of *HPS5*, requiring further studies to determine its structure and clinical relevance (Figure S2). The OGM result of P13 is shown in Figure 8.

## 6. Variants of Uncertain Significance

**Figure 9:**
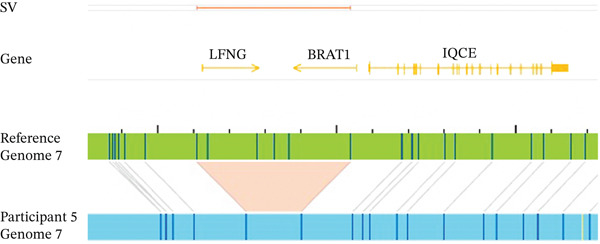
The genome browser image of P5. The deletion overlaps within *LFNG* and *BRAT1*. Horizontal green and blue bars represent the reference and the participant genome of Chromosome 7, respectively. The red line in the SV track indicates the heterozygous variant.

In P5, OGM detected a 27,527‐bp paternal heterozygous deletion in 7p22.3 involving *LFNG* and *BRAT1*. Although *LFNG* and *BRAT1* have been discussed as candidate genes in individuals with Asperger‐like features, neurodevelopmental delay, and behavioral abnormalities, the available evidence remains insufficient to establish pathogenicity [34, 35]. The OGM result of P5 is shown in Figure 9.

**Figure 10:**
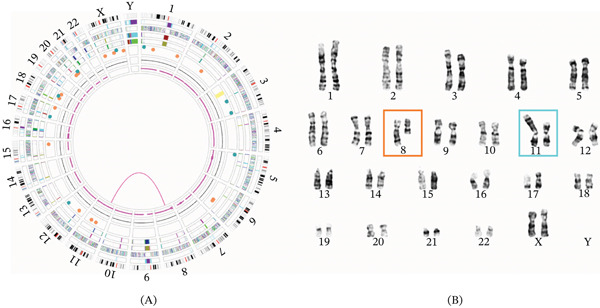
(A) The circos plot image of P6. The red line indicates the balanced t(8;11)(q11.21;p13). (B) Karyotype of P6. The orange rectangle indicates Chromosome 8 and the turquoise rectangle indicates Chromosome 11. The karyotype shows t(8;11)(q11.21;p13).

In P6, OGM identified a balanced t(8; 11)(q11.21; p13), with *SNTG1* located at the breakpoint. Because only limited evidence links *SNTG1* disruption to neurodevelopmental phenotypes, the clinical significance of this rearrangement remains uncertain [36]. The OGM and karyotype results of P6 are shown in Figure 10.

**Figure 11:**
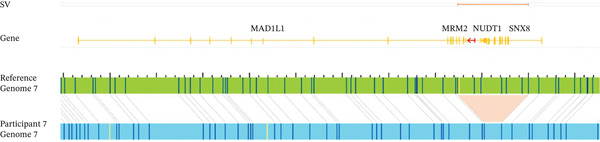
The genome browser image of P7. The deletion overlaps within *MAD1L1, MRM2, NUDT1*, and *SNX8*. Horizontal green and blue bars represent the reference and the participant genome of Chromosome 7, respectively. The red line in the SV track indicates the heterozygous variant.

In P7, OGM detected a 57,515‐bp heterozygous deletion in 7p22.3 involving *MAD1L1, MRM2, NUDT1,* and *SNX8*. Previous reports have suggested a possible association between 7p22.3 deletions and neurodevelopmental or psychiatric phenotypes, but a definitive genotype–phenotype correlation has not been established [37, 38]. The OGM result of P7 is shown in Figure 11.

**Figure 12:**
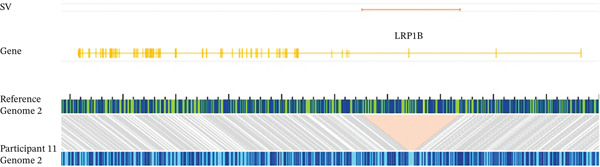
The genome browser image of P11. The deletion overlaps within *LRP1B*. Horizontal green and blue bars represent the reference and the participant genome of Chromosome 2, respectively. The red line in the SV track indicates the heterozygous variant.

In P11, OGM detected a 345,944‐bp heterozygous deletion in 2q22.1q22.2 involving *LRP1B*. Although *LRP1B* has been reported in ASD‐related molecular studies, the clinical relevance of this heterozygous deletion remains uncertain [39]. The OGM result of P11 is shown in Figure 12.

**Figure 13:**
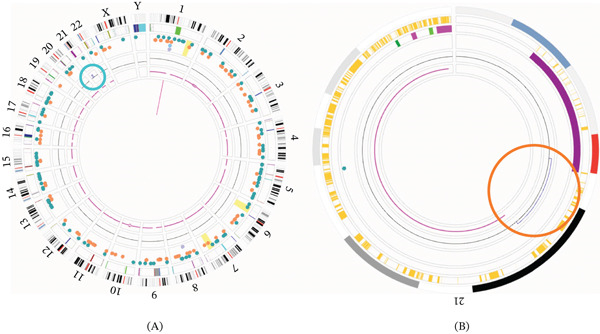
(A) The circos plot image of P12. The turquoise ring indicates the copy number gain in 21q11.1q21.1. (B) Chromosome 21 is shown in the circos plot. The orange ring indicates the copy number gain in 21q11.1q21.1 in an enlarged view.

In P12, OGM detected an unconfirmed 5,851,786‐bp duplication in 21q11.1q21.1 involving *NRIP1*. Given the lack of orthogonal confirmation and the limited evidence linking *NRIP1* dosage to ASD, this finding should be interpreted cautiously as an OGM‐only VUS requiring further validation [40, 41]. The OGM results of P12 are shown in Figure 13.

## 7. Discussion

The diagnostic contribution of OGM in this cohort should be interpreted in light of the phenotypic composition of the study population. Most individuals in our cohort had complex ASD with DD/ID and/or additional neurological, congenital, dysmorphic, or systemic features. All confirmed or provisional P/LP findings were detected in this complex ASD subgroup, whereas no P/LP finding was identified among individuals with ASD without documented DD/ID or other major neurodevelopmental findings. This observation is consistent with the broader genetic testing literature showing that diagnostic yield is generally higher in individuals with ASD accompanied by DD/ID, congenital anomalies, epilepsy, or dysmorphic features than in individuals with isolated ASD. Therefore, our findings support the potential utility of OGM in selected individuals with complex ASD, rather than as a standalone first‐tier test for all individuals with ASD.

The novelty of this study does not reside in the use of a new OGM workflow, as de novo assembly and targeted repeat‐expansion pipelines are established components of OGM analysis. Rather, the value of the present study lies in the clinical and genomic characterization of OGM‐detected findings in an ASD‐focused cohort from Türkiye. The study highlights several clinically relevant categories of findings, including pathogenic CNVs, balanced and unbalanced rearrangements, sex chromosome aneuploidy, intragenic homozygous deletions, and *FMR1* full‐mutation repeat expansion with suspected mosaicism. These observations support the complementary role of OGM in selected individuals with complex ASD, particularly when structural, chromosomal, or repeat‐expansion mechanisms are suspected or incompletely resolved by conventional methods.

Larger multisite OGM studies in constitutional postnatal cohorts have already demonstrated the broader technical and clinical utility of OGM; therefore, our study should be interpreted as an ASD‐focused, clinically detailed contribution from a selected Turkish cohort rather than as a large‐scale diagnostic‐yield study.

Despite the increased diagnostic yield of sequencing technologies in ASD, prevailing guidelines, such as the 2020 American Academy of Pediatrics (AAP), still recommend CMA as the first‐tier diagnostic test for ASD, highlighting the clinical relevance of SVs and CNVs [13]. The main methods used to analyze SVs and CNVs in ASD include karyotyping, FISH, MLPA, CMA, fragment analysis, and sequencing technologies. Because of its low resolution, karyotyping may miss a significant number of SVs. FISH and MLPA are targeted assays and cannot provide genome‐wide analysis. In contrast, OGM provides complementary advantages over several conventional cytogenetic methods by offering higher resolution for many SV classes, enabling the detection of balanced translocations and inversions, and supporting more precise breakpoint localization. In our cohort, OGM identified SVs and CNVs that would have required multiple conventional assays to resolve, supporting its role as a complementary cytogenomic method in selected individuals with complex ASD/NDD. However, OGM has limitations in detecting Robertsonian translocations and other centromeric fusions, for which karyotyping and FISH may remain useful.

Compared with CMA, OGM may provide additional advantages in selected contexts, including the detection of balanced translocations and inversions, improved breakpoint localization, more accurate sizing of some SVs, and assessment of selected repeat expansions. In P10, the *TCF4* deletion was not resolved by CMA in our analysis, whereas OGM estimated the deletion size as 87,573 bp, closely matching the 87,612‐bp deletion detected by WGS. In addition, unlike CMA, OGM can provide repeat‐number information for selected repeat‐expansion loci and may detect full‐mutation‐range expansions beyond the upper detection thresholds of some commercial repeat‐analysis kits [42].

NGS‐based sequencing technologies have the advantage of providing sequence‐level information in addition to SV analysis. These technologies, particularly WES, are currently used in routine clinical practice for ASD evaluation; however, they have limitations in detecting large and complex SVs, repeat expansions, and variants in repetitive genomic regions. WES is also largely restricted to coding regions and splice junctions and does not comprehensively assess regulatory regions such as promoters, enhancers, silencers, and insulators. Although not yet routinely used in many clinical settings, long‐read third‐generation sequencing technologies are emerging as useful approaches for elucidating the genetic etiology of ASD and can assess methylation status, unlike OGM. However, studies have shown that OGM can detect SVs missed by both second‐ and third‐generation sequencing technologies [43, 44]. This advantage is partly attributable to the substantially longer molecules analyzed by OGM compared with the reads generated by sequencing‐based approaches. In contrast, long‐read sequencing generally requires specialized instrumentation, substantial computational analysis, and customized bioinformatics workflows, which may increase complexity and cost [45].

Another challenge in the molecular diagnosis of ASD is the presence of segmental duplications and pseudoautosomal regions, which can complicate accurate variant detection and interpretation. Segmental duplications occur in genomic regions associated with several well‐characterized NDDs, including Williams–Beuren syndrome (OMIM#194050), Prader–Willi syndrome (OMIM#176270), and distal chromosome 22q11.2 deletion syndrome (OMIM#611867). Our study suggests that OGM may provide an advantage in analyzing such regions by reducing alignment‐related noise and improving variant interpretation in contexts where traditional sequencing‐based methods may yield false‐positive or false‐negative results. However, manual verification using the Access genome browser remains essential, and clinically relevant findings should be confirmed by orthogonal methods whenever possible.

For *FMR1* repeat‐expansion analysis, OGM may provide complementary value by enabling the detection of full‐mutation repeat expansions together with genome‐wide assessment of SVs, including CNVs, within the same workflow. Moreover, OGM can identify full‐mutation‐range expansions (> 200 repeats) and may provide supportive evidence for mosaic *FMR1* alleles when interpreted together with de novo assembly and orthogonal repeat testing [42]. As observed in P13, however, mosaic alleles with closely spaced repeat numbers may go undetected by the EnFocus Fragile X pipeline, emphasizing the need to integrate the de novo assembly pipeline for allele discrimination. By analogy with the combined pipeline strategy used in FSHD analysis, evaluation of both pipelines may also be useful in *FMR1* analyses [46]. In addition, OGM cannot reliably detect premutation‐range repeat expansions because they are below its reliable detection threshold; therefore, alternative validated methods remain necessary for premutation analysis. *FMR1* repeat testing is recommended for individuals presenting with DD/ID and/or ASD [47]. Overall, our findings highlight the complementary value of OGM in selected individuals with complex ASD/NDD, particularly when full‐mutation repeat expansions and SVs or CNVs are considered within the same cytogenomic workflow.

Our findings suggest that OGM may be considered as part of an integrated diagnostic workflow for selected individuals with complex ASD/NDD, particularly in cases with suspected SVs, chromosomal rearrangements, repeat expansions, or unresolved molecular etiology after conventional genetic testing. As discussed above, OGM can provide additional information in genomic contexts where other methods may be limited, including breakpoint resolution, balanced translocations, inversions, complex rearrangements, and selected repeat expansions. By clarifying variants that may be undetected, incompletely resolved, or difficult to interpret using conventional approaches, OGM may reduce diagnostic uncertainty and contribute to clinical interpretation, as has been reported in constitutional genetic disorders and hematological malignancies [48, 49]. Although OGM has been proposed as a potential first‐tier cytogenomic test in some settings because it can consolidate several cytogenetic analyses into a single assay, our data support a more cautious interpretation in ASD [50]. Because OGM does not reliably detect SNVs, small indels, or premutation‐range repeat expansions, it should not be considered a standalone diagnostic test for ASD. Rather, it is best positioned as a complementary cytogenomic approach alongside WES/WGS, CMA, karyotyping, targeted repeat analysis, or long‐read sequencing, depending on the clinical indication and available resources. This positioning is also consistent with studies reporting that OGM may increase diagnostic yield in unsolved NDD cases after negative or inconclusive sequence‐based testing [51–53], with reported incremental diagnostic contributions ranging from 4.5% to 10.6% in selected unsolved NDD cohorts [15, 51, 54].

This study has several limitations. First, the cohort size was modest (*n* = 34), which limits statistical power and prevents robust estimation of the diagnostic yield of OGM in ASD. Therefore, our findings should not be interpreted as providing a definitive or population‐level diagnostic yield for ASD. Rather, this study provides a clinically detailed description of OGM‐detected SVs, including CNVs, chromosomal abnormalities, and repeat‐expansion findings in a selected cohort enriched for complex ASD and additional neurodevelopmental features. Second, most individuals in our cohort had ASD accompanied by DD/ID and/or additional neurological, congenital, dysmorphic, or systemic findings. All confirmed or provisional P/LP findings were identified in this complex ASD subgroup, whereas no P/LP finding was detected among individuals with ASD without documented DD/ID or other major neurodevelopmental findings. This phenotypic composition limits the generalizability of our findings to individuals with isolated ASD.

Third, OGM has important technical limitations. It does not reliably detect SNVs, small indels, or small repeat expansions below its reliable detection threshold, including premutation‐range repeat expansions. Therefore, OGM should not be considered a standalone genetic test for ASD and should be interpreted as complementary to sequence‐based approaches such as WES, WGS, and, in selected settings, long‐read sequencing. Fourth, the retrospective design limited the availability of standardized IQ scores, adaptive‐functioning measures, and uniform developmental assessments for all individuals, restricting our ability to stratify diagnostic findings by ID severity or developmental profile. Fifth, because the study was conducted at clinical genetics centers, the cohort may be subject to ascertainment bias, with enrichment for individuals with complex ASD, dysmorphic features, congenital anomalies, epilepsy, or prior negative genetic testing. Therefore, the observed diagnostic contribution of OGM may be higher than would be expected in an unselected ASD population.

Finally, although most OGM‐detected findings were confirmed by orthogonal methods, two findings could not be independently validated. These OGM‐only findings were explicitly marked as unconfirmed and interpreted cautiously, and they should not be regarded as definitive clinically actionable findings without further validation. Future studies should include larger, prospective, multicenter cohorts with systematic phenotyping, standardized cognitive and adaptive‐functioning assessments, predefined inclusion criteria, and complete orthogonal validation of clinically relevant findings. Integrated diagnostic strategies combining OGM with WES/WGS and, where appropriate, long‐read sequencing may further clarify the complementary role of OGM in ASD and broader NDD cohorts.

## 8. Conclusion

In this study, we evaluated the contribution of OGM in a selected cohort of individuals with ASD, predominantly enriched for complex ASD with DD/ID and/or additional neurodevelopmental, neurological, congenital, or syndromic features. OGM identified confirmed P/LP findings in 7 of 34 individuals, with one additional unconfirmed OGM‐only duplication interpreted as provisional P; all of these individuals had complex ASD. These results support the use of OGM as a complementary cytogenomic approach in selected individuals with complex ASD/NDD, particularly for detecting SVs, complex rearrangements, chromosomal abnormalities, and *FMR1* full‐mutation repeat expansions that may not be fully resolved by conventional methods. Larger prospective studies with systematic phenotyping and complete orthogonal validation are needed to define the precise clinical role of OGM within integrated ASD genetic testing algorithms.

## Author Contributions

M.B.M., Ö.B.G.Ö., Ö.Ö., and F.K. contributed to the conception and design of the study. M.B.M., F.K., F.De., S.S., A.G.B.T., E.T., H.Ö., M.E., H.G., and S.K. contributed to participant recruitment, clinical assessment, and interpretation of clinical data. M.B.M., Ö.B.G.Ö., Ö.Ö., F.Du., N.S., K.B., and S.E. performed and interpreted the OGM/genomic analyses. M.B.M., Ö.B.G.Ö., Ö.Ö., and F.K. drafted the manuscript. F.D., S.S., A.G.B.T., E.T., H.Ö., M.E., H.G., and S.K. critically revised the manuscript.

## Funding

No funding was received for this manuscript.

## Disclosure

All authors read and approved the final version of the manuscript and agree to be accountable for the work.

## Ethics Statement

The study protocol was approved by the Harran University Clinical Research Ethics Committee (HRÜ/24.14.22). Written informed consent for genetic testing and for the use of anonymized clinical and genomic data for research and publication was obtained from the parents or legal guardians of all participants.

## Conflicts of Interest

Mehmet Burak Mutlu, Fatma Demiryılmaz, Nurcan Silahtarlıoğlu, Kader Bilgil, and Sümeyye Elma are employed by a genetic diseases evaluation company that distributes OGM technology in Türkiye. The other authors declare no conflicts of interest.

## Supporting information


**Supporting Information** Additional supporting information can be found online in the Supporting Information section. Table S1. The sociodemographic information and clinical features of participants. Supporting Information Figure S1: The fragment analysis images of Participant 13. Supporting Information Figure S2: The genome browser image of Participant 13.

## Data Availability

The data that support the findings of this study are available on request from the corresponding author. The data are not publicly available due to privacy or ethical restrictions.
