# Supplementary material for: Structural Variant and Repeat Expansion Findings Identified by Optical Genome Mapping in Complex Autism Spectrum Disorder With Concomitant Neurodevelopmental Disorders
Source: Hum Mutat. 2026 Jun 11;2026:3130383. doi: 10.1155/humu/3130383 (PMC13255017; doi:10.1155/humu/3130383)
Supplement: Supplementary file 1 — Supporting Information Additional supporting information can be found online in the Supporting Information section. Table S1. The sociodemographic information and clinical features of participants. Supporting Information Figure S1: The fragment analysis images of Participant 13. Supporting Information Figure S2: The genome browser image of Participant 13. [file HUMU-2026-3130383-s001.docx]

**Structural Variant and Repeat Expansion Findings Identified by Optical Genome Mapping in Complex Autism Spectrum Disorder with Concomitant Neurodevelopmental Disorders**

| **Participants** | **Sex** | **Age** | **Psychiatric Features** | **Other Clinical Features** |
| --- | --- | --- | --- | --- |
| **14** | F | 2 | ASD | DD/ID, speech delay, delayed ability to walk, epilepsy, dysmorphic features (synophrys), microcephaly, corpus callosum agenesis, swallowing problem |
| **15** | F | 3 | ASD | DD/ID, inability to speak and walk, hypotonia |
| **16** | F | 4 | ASD | DD/ID, speech delay, delayed ability to walk |
| **17** | F | 4 | ASD | DD/ID, hypotonia, inflammatory intestinal disease |
| **18** | M | 9 | ASD | DD/ID, speech delay, delayed ability to walk, tall stature, dysmorphic features (macrocephaly, long face, prominent forehead) |
| **19** | M | 10 | ASD | DD/ID, inability to speak, delayed ability to walk, gait abnormality, EEG abnormality, epilepsy? |
| **20** | F | 9 | ASD | DD/ID, inability to speak and walk, spasticity, hypotonia, epilepsy, dysmorphic features (midface hypoplasia, abnormal dermatoglyphics) |
| **21** | M | 5 | ASD | DD/ID, speech delay, delayed ability to walk, hypotonia |
| **22** | M | 4 | ASD | Spastic paresis, gait abnormality |
| **23** | F | 5 | ASD | DD/ID, speech delay |
| **24** | M | 4 | ASD | Infantile spasms |
| **25** | F | 3 | ASD | DD/ID, speech delay, delayed ability to walk, microcephaly, dysmorphic features (epicanthus, low-set, small ears, strabismus) |
| **26** | F | 4 | ASD | DD/ID, speech delay, delayed ability to walk, epilepsy |
| **27** | F | 11 | ASD | DD/ID, speech delay, delayed ability to walk, microcephaly, dysmorphic features (facial asymmetry, strabismus, diastema), atrial septal defect, high pain tolerance, chronic constipation |
| **28** | F | 8 | ASD, ADHD | DD/ID, inability to speak, delayed ability to walk |
| **29** | M | 6 | ASD | DD/ID, speech delay, delayed ability to walk, epilepsy, hypotonia |
| **30** | F | 5 | ASD, ADHD | Epilepsy |
| **31** | M | 4 | ASD | DD/ID, speech delay, epilepsy, hypotonia |
| **32** | F | 5 | ASD | DD/ID, inability to speak, delayed ability to walk, scoliosis, tall stature, growth abnormality |
| **33** | F | 17 | ASD, ADHD, Anxiety disorder | DD/ID, speech delay, delayed ability to walk, abnormality of speaking (lisp) |
| **34** | F | 12 | ASD | DD/ID, inability to speak and walk, cerebral -cerebellar atrophy |

**Supplementary Table 1:** The sociodemographic information and clinical features of participants. F: female, M: male; DD/ID: developmental delay/intellectual disability, ASD: autism spectrum disorder, ADHD: attention deficit hyperactivity disorder, OCD: obsessive compulsive disorder


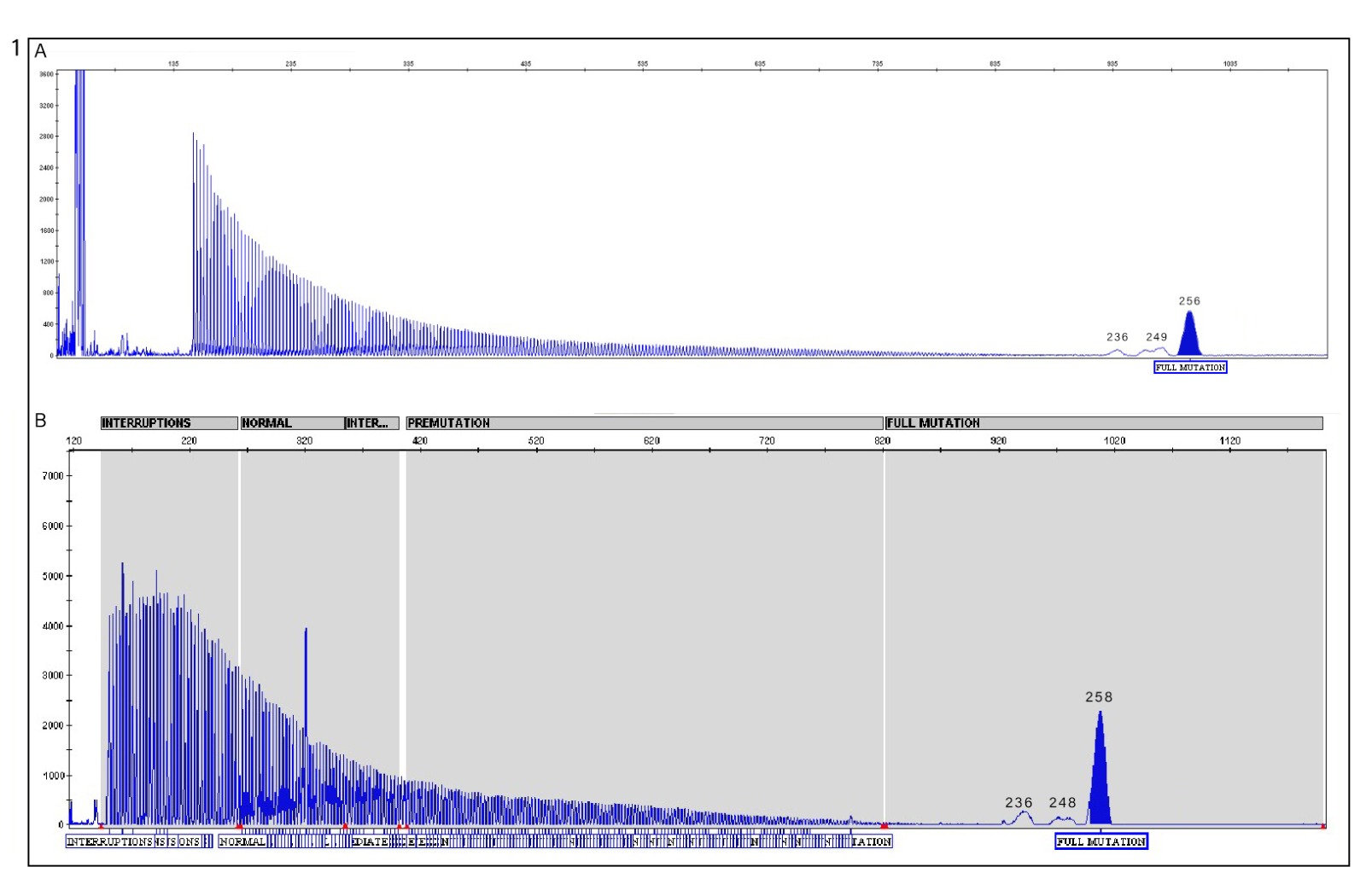


**Supplementary Figure 1.** Fragment analysis images of participant 13. **A)** Analysis performed using the Adellgene® Fragile X diagnostic kit. **B)** Analysis performed using the LabGscan™ FRAXA PCR diagnostic kit. Both assays supported full-mutation-range *FMR1* alleles with closely spaced repeat numbers, consistent with mosaicism. Kit-specific repeat-size variability should be considered when interpreting repeat-number estimates.


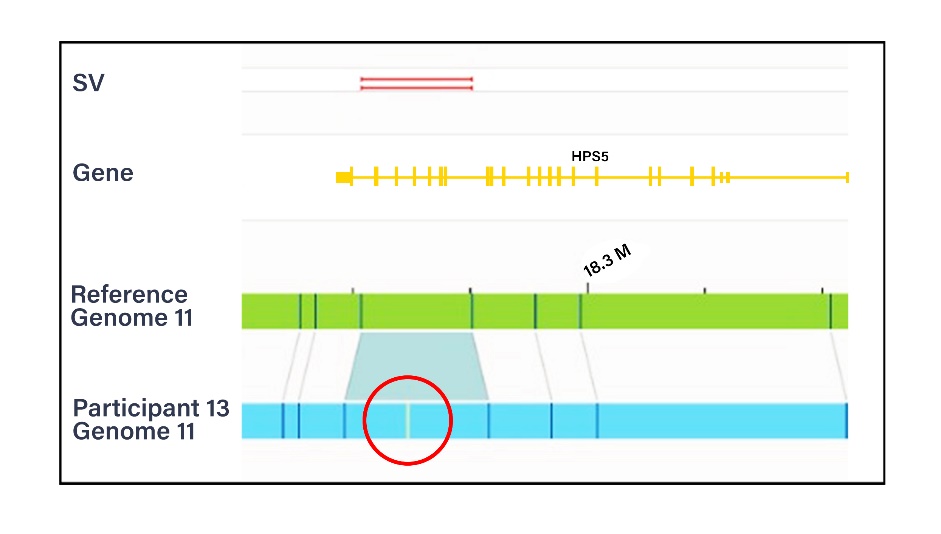


**Supplementary Figure 2:** The genome browser image of participant 13. The insertion overlaps within *HPS5.* The orphan label in the red circle is located between exon 18 and 19 (NM_181508.1). Horizontal long green and blue bars represent the reference and the participant genomes of chromosome 15, respectively. Two lines in the SV track indicate the insertion call.
